# New evidence for the cerebellar involvement in personality traits

**DOI:** 10.3389/fnbeh.2013.00133

**Published:** 2013-10-02

**Authors:** Eleonora Picerni, Laura Petrosini, Fabrizio Piras, Daniela Laricchiuta, Debora Cutuli, Chiara Chiapponi, Sabrina Fagioli, Paolo Girardi, Carlo Caltagirone, Gianfranco Spalletta

**Affiliations:** ^1^I.R.C.C.S. Santa Lucia FoundationRome, Italy; ^2^Department of Psychology, Faculty of Medicine and Psychology, University “Sapienza” of RomeRome, Italy; ^3^NESMOS Department, Faculty of Medicine and Psychology, University “Sapienza” of RomeRome, Italy; ^4^Department of Neuroscience, Tor Vergata UniversityRome, Italy

**Keywords:** Voxel-based morphometry, mean diffusivity, fractional anisotropy, novelty seeking, individual differences, temperamental traits

## Abstract

Following the recognition of its role in sensory-motor coordination and learning, the cerebellum has been involved in cognitive, emotional, and even personality domains. This study investigated the relationships between cerebellar macro- and micro-structural variations and temperamental traits measured by Temperament and Character Inventory (TCI). High resolution T1-weighted, and Diffusion Tensor Images of 100 healthy subjects aged 18–59 years were acquired by 3 Tesla Magnetic Resonance scanner. In multiple regression analyses, cerebellar Gray Matter (GM) or White Matter (WM) volumes, GM Mean Diffusivity (MD), and WM Fractional Anisotropy (FA) were used as dependent variables, TCI scores as regressors, gender, age, and education years as covariates. Novelty Seeking scores were associated positively with the cerebellar GM volumes and FA, and negatively with MD. No significant association between Harm Avoidance, Reward Dependence or Persistence scores and cerebellar structural measures was found. The present data put toward a cerebellar involvement in the management of novelty.

## Introduction

Connected first to motor and then cognitive functions, the cerebellum has been involved also in new domains, as emotional regulation and affective processing (Schmahmann and Sherman, 1998; Paradiso et al., 1999; Schmahmann et al., 2007; Timmann and Daum, 2007). Namely, it has been demonstrated that the cerebellum is not only the coordinator of motor function but it is implicated in timing and monitoring functions (Ivry and Spencer, 2004) integration of somatic and visceral information (Zhu et al., 2006) spatial and executive functions, and linguistic processing (De Smet et al., 2013). Anatomo-clinical analyses indicated the cerebellum as a critical neuro-modulator of intellect and mood and revealed that the posterior vermis, the so-called limbic cerebellum is mainly involved in the regulation of emotion and affect (Schmahmann, 1991, 1996, 2004; Stoodley and Shmahmann, 2010). A range of cognitive, linguistic and affective deficits and personality changes (cerebellar cognitive-affective syndrome) are described in subjects with lesions of the posterior lobe and vermis (Schmahmann and Sherman, 1998). The core features of the syndrome are executive dysfunctions such as disturbances in planning, set-shifting, abstract reasoning and working memory, visuo-spatial deficits, such as impaired visuo-spatial organization and memory, language symptoms, as agrammatism and anomia, and behavioral-affective disturbances, consisting of blunting of affect or disinhibited and inappropriate behavior (Schmahmann and Sherman, 1998). Magnetic Resonance Imaging (MRI) studies showed structural or functional abnormalities of the cerebellum in patients with personality, depression or anxiety disorders (Pillay et al., 1997; De Bellis and Kuchibhatla, 2006; Fitzgerald et al., 2008; Baldaçara et al., 2011). Even data obtained in healthy subjects indicated reduced capacities of emotional regulation following inhibitory repetitive transcranial magnetic stimulation to the cerebellum (Schutter and Van Honk, 2009). The functional topographic organization of cerebellum indicates that the motor functions are represented in the anterior cerebellum (lobules I–V) and the sensory-motor functions in lobule VIII; the cognitive functions are supported by the posterior cerebellum (lobules VI and VII, including Crus I and II and lobule VIIb); the emotional regulation has as anatomical substrate the posterior vermis (lobules VI, VII, and VIII, Crus I and II, lobule VIIb) (Stoodley and Shmahmann, 2010; Stoodley et al., 2012). Such a functional topography is a consequence of the differential arrangement of cerebellar connections with spinal cord, brainstem, and cerebral hemispheres. These loops are organized such that cerebellar regions that receive input from a given area relay output back to the same area, thus forming parallel segregated circuits. This organization, however, detailed it may be, does not take into account a cerebellar involvement in the personality traits.

Since the several personality indices are rooted in behavior and inescapably have a strong action component, the link between the cerebellum and personality traits is intriguing but not meaningless. By combining various neuroimaging techniques and personality measures in healthy subjects, several studies advanced the involvement of the cerebellum in some dimensions of personality models. Within these, while Eysenck's model (1967, 1981) postulates three biologically-based independent dimensions, Extraversion, Neuroticism and Psychoticism, Cloninger's model (1986) proposes four genetically- and biologically-based independent temperamental dimensions, Novelty Seeking (NS), Harm Avoidance (HA), Reward Dependence (RD) and Persistence (P). Cerebellar perfusion was correlated positively with NS or Extraversion and negatively with HA (O'Gorman et al., 2006). Positive associations between pattern of synchronous cerebellar neuronal activity and Extraversion were also described (Wei et al., 2011). Furthermore, recent results showed that White Matter (WM) and Gray Matter (GM) cerebellar volumes negatively covaried with neurotic personality traits (Schutter et al., 2012). Analyzing the relationships between the amplitude of spontaneous low-frequency oscillations and personality traits by using resting-state functional MRI, behavioral inhibition appeared to be correlated negatively with the cerebellum and positively with the frontal gyrus (Kunisato et al., 2011).

Recently, by using a Region of Interest (ROI)-based approach, in a large sample of healthy subjects we reported WM and GM cerebellar volumes associated positively with NS and negatively with HA (Laricchiuta et al., 2012), while no significant association was found between cerebellar volumes and RD and *P* scores.

Given such a volumetric approach prevented the precise localization of the cerebellar regions involved in personality traits, the present study analyzed the associations between specific cerebellar regions with the four temperamental traits by Cloninger. To detect macro-structural organization, Voxel-Based Morphometry (VBM) analysis was used, while to detect micro-structural organization, Diffusion Tensor Imaging (DTI) scan protocol was employed.

DTI measures the diffusion of water molecules through tissues, detects brain micro-structural variations, and provides physiological information not available by using conventional MRI (Basser and Pierpaoli, 1996). Indeed, water molecules move in the brain interacting with many tissue components, such as cell membranes, fibers or macromolecules. The indirect observation of these displacements provides valuable information on the microscopic obstacles encountered by diffusing water molecules, and in turn, on size of the pores between cells as well as structure, density, surface and orientation of cells (Le Bihan, 2007).

Among DTI indices we used the Mean Diffusivity (MD) for GM and the Fractional Anisotropy (FA) for WM. In particular, MD measures the average extent of water diffusion, providing information on restrictions and boundaries (e.g., high density of cells) that water molecules encounter. If these obstacles have coherent alignment, on average the water tends to diffuse more along a certain axis. Although it has been traditionally used to study WM features, recent studies have indicated that MD allows studying even GM variations (Müller et al., 2007; Piras et al., 2010, 2011; Spalletta et al., 2012). Unlike WM, GM is less organized in orientation. This lack of coherent orientation limits the use of MD to the GM structures that exhibit high directionality in diffusion because of the presence of coherent WM nearby. Cerebellar GM meets such a condition. In several pathological states, the altered GM cytoarchitecture causes enlargement of the extracellular space that results in the MD increase (Syková, 2004). In physiological states, changes in efficacy of synaptic transmission influence extra-cellular water diffusion parameters (Sykovà and Nicholson, 2008). Accordingly, low MD values of GM nuclei have been linked to improved cognitive performances (Kantarci et al., 2011; Piras et al., 2011; Spalletta et al., 2012). Very recently, we found increased MD in the bilateral putamen associated with high HA personality trait (Laricchiuta et al., 2013).

FA measures anisotropy of water diffusion and it is positively linked to fiber density, axonal diameter and myelination in WM. Low FA values stand for isotropic diffusion (i.e., unrestricted in all directions), while high FA values indicate diffusion fully restricted along one axis. Thus, FA is high when the density of the ordered structures (axonal fibers) is high (Pierpaoli et al., 1996).

As a further step to investigate the neurobiological bases of personality, in the present research, we hypothesize that the *cerebellar involvement* already described for motor, cognitive and affective functions may work also for temperamental traits. To this aim, the present study investigated the associations between scores of Temperament and Character Inventory (TCI) by Cloninger (1986) and cerebellar macro- (VBM) and micro- (MD and FA) structural variations in a large sample of differently aged healthy subjects, by using a 3 Tesla high-resolution structural MRI and a DTI protocol.

## Materials and methods

### Ethics statement

The study was approved by the Local Ethics Committee of the I.R.C.C.S. Santa Lucia Foundation and written consent was obtained from all participants after full explanation of study procedures.

### Participants

A sample of 100 healthy subjects (43 males; mean age ± *SD*: 48.5 ± 3.5 years; range: 18–59) was recruited for the study. Educational level ranged from an eighth grade to a post-graduate degree (mean education years ± *SD*: 16.11 ± 3.18; range: 8–24). All participants were right-handed as assessed with the Edinburgh Handedness Inventory (Oldfield, 1971). Seventy-five out of the 100 subjects had also been included in our previous study (Laricchiuta et al., 2012), while 25 new subjects were added. Out of 125 subjects of the previous study, fifty participants were not included in the present research since their DTI images exhibited bad coverage and venetian blind artifacts at the level of the cerebellum. In the present study the inclusion criteria were age between 18 and 70 years and suitability for MRI scanning. Exclusion criteria included (i) suspicion of cognitive impairment or dementia based on Mini Mental State Examination (MMSE) (Folstein et al., 1975) scores = 24 (Measso et al., 1993), and confirmed by clinical neuropsychological evaluation by using the Mental Deterioration Battery (Carlesimo et al., 1996) and the NINCDS-ADRDA criteria for dementia (McKhann et al., 1984); (ii) subjective complaint of memory difficulties or of any other cognitive deficit, regardless of interference with daily activities; (iii) major medical illnesses, e.g., diabetes (not stabilized), obstructive pulmonary disease, or asthma; hematologic and oncologic disorders; pernicious anemia; clinically significant gastrointestinal, renal, hepatic, endocrine, or cardiovascular system diseases; newly treated hypothyroidism; (iv) current or reported psychiatric (assessed by SCID-I and the SCID-II) (First et al., 1997, 2002) or neurological (assessed by clinical neurological evaluation) disorders (e.g., schizophrenia, mood disorders, anxiety disorders, stroke, Parkinson's disease, seizure disorder, head injury with loss of consciousness, and any other significant mental or neurological disorder); (v) known or suspected history of alcoholism or drug dependence and abuse, evaluated by structured interviews (SCID I or SCID II) (First et al., 1997, 2002); (vi) MRI evidence of focal parenchymal abnormalities or cerebro-vascular diseases: for each subject, a trained neuroradiologist and a neuropsychologist expert in neuroimaging co-inspected all the available clinical MRI sequences (i.e., T1 and T2-weighted and FLAIR images) to ensure that the subjects were free from structural brain pathologies and vascular lesions (i.e., FLAIR or T2-weighted hyper-intensities and T1-weighted hypo-intensities).

### Image acquisition

Participants underwent an imaging protocol that included standard clinical sequences (FLAIR, DP-T2-weighted), a volumetric whole-brain 3D high-resolution T1-weighted sequence, and a DTI scan protocol, performed with a 3T Allegra MR imager (Siemens, Erlangen, Germany). Volumetric whole-brain T1-weighted images were obtained in the sagittal plane by using a modified driven equilibrium Fourier transform (MDEFT) sequence (Echo Time/Repetition Time -TE/TR- = 2.4/7.92 ms, flip angle 15°, voxel-size 1 × 1 × 1 mm^3^).

Diffusion volumes were acquired by using echo-planar imaging (TE/TR = 89/8500 ms, bandwidth = 2126 Hz/vx; matrix size 128 × 128; 80 axial slices, voxel size 1.8 × 1.8 × 1.8 mm^3^) with 30 isotropically distributed orientations for the diffusion-sensitizing gradients at one *b*-value of 1000 s·mm^2^ and two *b* = 0 images. Scanning was repeated three times to increase the signal-to-noise ratio.

All planar sequence acquisitions were obtained in the plane of the anterior-posterior commissure line. Since the posterior cranial fossa usually falls at the lower limit of the field of view, particular care was taken to center subject's head in the head coil, to avoid possible magnetic field dishomogeneities or artifacts at cerebellar level.

### Image processing

T1-weighted and DTI images were submitted to several processing steps. First, T1-weighted images were processed and examined by using the SPM8 software (Wellcome Department of Imaging Neuroscience Group, London, UK; www.fil.ion.ucl.ac.uk/spm), specifically the VBM8 toolbox (http://dbm.neuro.uni-jena.de/vbm.html) running in Matlab 2007b (MathWorks, Natick, MA, USA). The toolbox extends the unified segmentation model (Ashburner and Friston, 2005) consisting of MRI field intensity inhomogeneity correction, spatial normalization and tissue segmentation at several pre-processing steps to further improve data quality. Initially, to increase the signal-to-noise ratio, an optimized block wise nonlocal-means filter was applied to the MRI scans by using the Rician noise adaption (Wiest-Daesslé et al., 2008). Then, an adaptive maximum *a posteriori* segmentation approach extended by partial volume estimation was employed to separate the MRI scans into GM, WM and cerebro-spinal fluid. The segmentation step was finished by applying a spatial constraint to the segmented tissue probability maps based on a hidden Markow Random Field model to remove isolated voxels which unlikely were members of a certain tissue class and to close holes in clusters of connected voxels of a certain class, resulting in a higher signal-to-noise ratio of the final tissue probability maps. Then, the iterative high-dimensional normalization approach provided by the Diffeomorphic Anatomical Registration Through Exponentiated Lie Algebra (DARTEL) (Ashburner, 2007) toolbox was applied to the segmented tissue maps in order to register them to the stereotaxic space of the Montreal Neurological Institute (MNI). The tissue deformations were used to modulate participants' GM and WM maps to be entered in the analyses. Voxel values of the resulting normalized and modulated GM and WM segments indicated the probability (between 0 and 1) that a specific voxel belonged to the relative tissue. Finally, the modulated and normalized GM and WM segments were written with an isotropic voxel resolution of 1.5 mm^3^ and smoothed with a 6 mm Full-Width Half Maximum (FWHM) Gaussian kernel. The segmented, normalized, modulated and smoothed GM and WM images were used for analyses.

Subsequently, DTI images were processed by using FSL 4.1 (www.fmrib.ox.ac.uk/fsl/). Image distortions induced by eddy currents and head motion in the DTI data were corrected by applying a 3D full affine (mutual information cost function) alignment of each image to the mean no diffusion weighting (*b* = 0) image. After corrections, DTI data were averaged and concatenated into 31 (1 b0 + 30 b1000) volumes. A diffusion tensor model was fit at each voxel and maps of FA and MD were generated. FA was non-linearly transformed into standard space using the tool FNIRT (Andersson et al., 2007) and the transformation matrix was then applied to the MD maps which were subsequently smoothed by using a Gaussian kernel with a 6 mm FWHM.

Analyses were restricted to the cerebellum by using inclusive masks of cerebellar GM and WM, determined as follows: (i) GM mask was achieved by meaning all GM probability maps obtained in the VBM8 processing steps, thresholding the relative image to a value of 0.3 (i.e., removing all voxels having a probability to belong to GM lower or equal to 29%) and manually removing all the non-cerebellar structures by using the MNI-oriented atlas of the human brain (Automated Anatomical Labeling Atlas, AAL) (Tzourio-Mazoyer et al., 2002) as reference; (ii) similarly, WM mask was obtained by meaning all VBM8 WM probability maps, thresholding the relative image to a value of 0.3 and manually removing all the non-cerebellar structures by using the AAL template. To obtain the precise anatomical localization of results, we superimposed statistical maps onto the Diedrichsen's probabilistic atlas of the human cerebellum, which subdivides the cerebellum in 10 different regions (Diedrichsen et al., 2009).

### Personality assessment

TCI includes four temperamental scales (NS, HA, RD, and P) and represents a quantitative and reliable instrument to assess personality dimensions (Cloninger, 1986, 1987; Cloninger et al., 1993). TCI dimensions show adequate internal consistency coefficients (Martinotti et al., 2006). Subjects respond on a 5-point Likert scale ranging from 1 (definitively false) to 5 (definitively true) to enhance the sensitivity of measurement for subscales. The Italian TCI was validated by Fossati et al. (2001) in both clinical and non-clinical samples.

The NS scale consists of four sub-scales and refers to the tendency to action behavior. High NS scores stand for high tendency to exploratory activity in response to novelty, impulsive decision-making, extravagant approach to reward cues and quick loss of temper. The advantages of high NS are excitability, curiosity, enthusiasm and quick engagement with whatever is new and unfamiliar. Conversely, the disadvantages are indifference, lack of reflection and intolerance to monotony, anger, inconsistence in relationships and quick disengagement whenever a wish is frustrated.

The NS1 sub-scale (Exploratory Excitability vs. Stoic Rigidity) is related to the tendency vs. avoidance to explore unfamiliar places and situations; the NS2 sub-scale (Impulsiveness vs. Reflection) is related to the tendency vs. avoidance to be excitable, impressionistic and dramatic, to make decisions with incomplete information, to poorly control impulses; the NS3 sub-scale (Extravagance vs. Reserve) is related to the tendency vs. avoidance to be extravagant with money, energy and feelings and to live “at the edge”; the NS4 sub-scale (Disorderliness vs. Regimentation) is related to the tendency vs. avoidance to lose temper and to run away from whatever is frustrating, boring or uncomfortable.

The HA scale consists of four sub-scales and refers to a tendency to inhibition of behavior in response to signals of punishment or non-reward. High HA scores stand for high tendency to pessimistic worry in anticipation of problems, fear of uncertainty, shyness with strangers, rapid fatigability. Adaptive advantages of high HA are cautiousness and careful planning when hazard is likely. The disadvantages occur when hazard is unlikely but still anticipated, which leads to maladaptive inhibition and anxiety.

The HA1 sub-scale (Anticipatory worry vs. Uninhibited Optimism) is related to the avoidance vs. tendency to anticipate harm in unfamiliar situations; the HA2 sub-scale (Fear of Uncertainty) is related to the avoidance vs. tendency to tolerate potentially dangerous circumstances; the HA3 sub-scale (Shyness vs. strangers) is related to the avoidance vs. tendency to be assertive in social situations and to meet strangers; the last HA4 sub-scale (Fatigability vs. Vigor) is related to the avoidance vs. tendency to be sthenic and active, recovering quickly from illnesses or stress.

The RD scale consists of four sub-scales and refers to a tendency to the maintenance of behavior in response to cues of social reward. High RD scores stand for being tender-hearted, sensitive, dedicated, dependent, and sociable. Adaptive advantages of high RD are the sensitivity to social cues, which facilitates affectionate social relations and genuine care for others. The disadvantage is related to suggestibility and loss of objectivity frequently encountered with people who are excessively socially dependent.

The RD1 sub-scale (Sentimentality) is related to the tendency vs. avoidance to be deeply moved by sentimental appeals, personally experiencing what others are feeling; the RD2 sub-scale (Openness to warm communication) is related to the tendency vs. avoidance toward warm social relations, tending to show their emotions easily in presence of others; the RD3 sub-scale (Attachment vs. Detachment) is related to the tendency vs. avoidance to intimacy over privacy; the RD4 sub-scale (Dependence vs. Independence) is related to the tendency vs. avoidance to emotional support and approval from others.

The *P* scale consists of a single scale and refers to a tendency to the maintenance of behavior despite frustration, fatigue, and intermittent reinforcement. High *P* scores stand for hard-working, perseveration, ambitiousness and perception of frustration as a personal challenge. Adaptive advantage of high *P* is the use of behavioral strategy when rewards are intermittent but contingencies remain stable. The disadvantage is related to perfectionistic perseveration when the contingencies change rapidly.

In the present study, TCI scores (mean score ± *SD*: NS: 20.59 ± 4.51; HA: 13.67 ± 6.36; RD: 15.39 ± 3.81; P: 6.54 ± 4.14) have been separately correlated with cerebellar macro- and micro-structural indices.

### Statistical analysis

Parametric associations between TCI (total and sub-scales) scores and age or education years were analyzed by Pearson's product moment correlations (Fisher's *r* to *z*). The effect of gender was assessed by using unpaired 2sample *t*-test. Results of the analyses on the demographic variables were considered significant at *p* < 0.05 level.

Relationships between cerebellar GM and WM volumes and TCI (total and sub-scales) scores were tested at the voxel level by using SPM8 within the framework of the General Linear Model. Multiple-regression analyses were computed by singularly using the measures of GM (or WM) volumes, GM MD and WM FA as dependent variables, TCI scores as regressors, and age, education years and gender as covariates to control for the effects of the socio-demographic variables on the main effects. As for VBM results, we reported only relationships whose voxels were part of a spatially contiguous cluster size of a minimum of 10 voxels and that survived (*p* < 0.05) at the random field theory-based method from the Family-Wise Error (FWE) corrections (Nichols and Hayasaka, 2003). However, since to our knowledge this is the first study assessing the possible relationships between cerebellar micro-structural data and personality traits, to avoid the risk of false negatives, for DTI analyses we accepted an uncorrected statistical level of *p* < 0.0001.

**Figure 1:**
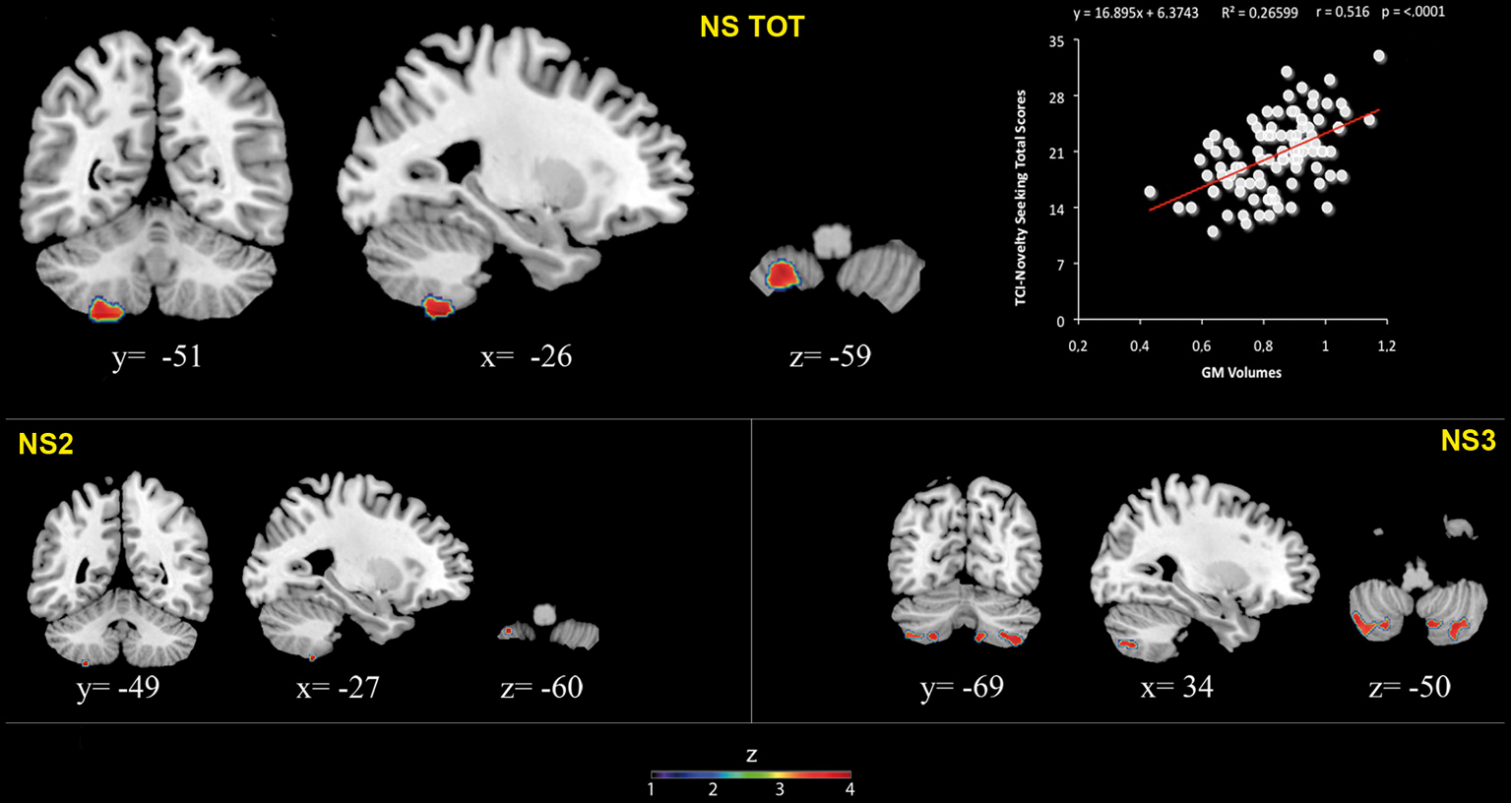
**Positive association between cerebellar gray matter volumes and Novelty Seeking (total and subscales) scores.** Coordinates are in Montreal Neurological Institute (MNI) space. Z above colorbar indicates normalized *t*-values. In figure left is left. Areas significantly associated with Novelty Seeking (NS) subscales in the voxel based analyses were used as regions of interest (ROI) to extract raw data and create scatterplot. Equation, *R*^2^, *r*, and *p*-values, as well as linear fit (solid red line) are reported.

**Figure 2:**
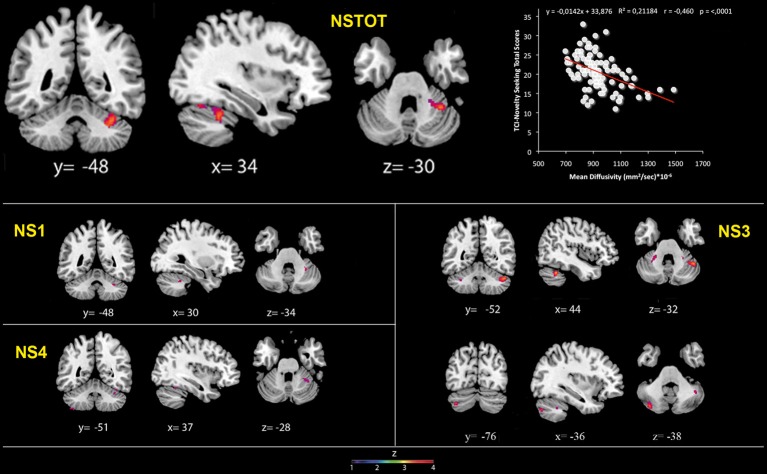
**Cerebellar gray matter Mean Diffusivity and Novelty Seeking (total and subscales) scores.** Coordinates are in Montreal Neurological Institute (MNI) space. Z above colorbar indicates normalized *t*-values. In figure left is left. Areas significantly associated with Novelty Seeking (NS) subscales in the voxel-based analyses were used as regions of interest (ROI) to extract raw data and create scatterplot. Equation, *R*^2^, *r*, and *p*-values, as well as linear fit (solid red line) are reported.

**Figure 3:**
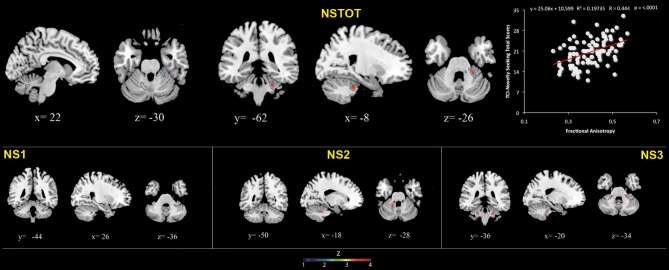
**Cerebellar white matter Fractional Anisotropy and Novelty Seeking (total and subscales) scores.** Coordinates are in Montreal Neurological Institute (MNI) space. Z above colorbar indicates normalized *t*-values. In figure left is left. Areas significantly associated with Novelty Seeking (NS) subscales in the voxel based analyses were used as regions of interest (ROI) to extract raw data and create scatterplot. Equation, *R*^2^, *r*, and *p*-values, as well as linear fit (solid red line) are reported.

Finally, to show the relationships between MRI data and personality measures, mean values of cerebellar areas significantly associated with TCI scores were extracted and used to create scatterplots and relative statistics shown in the Figures 1–3 of the main text and in the Supplementary Materials.

## Results

### Relationships between TCI scales and socio-demographic variables

There was a significant negative relationship between NS scores (total and NS1 and NS4) and age (NS: *r* = −0.25, *p* < 0.05; NS1: *r* = −0.25, *p* < 0.05; NS2: *r* = 0.11, *p* > 0.05; NS3: *r* = −0.18, *p* > 0.05; NS4: *r* = −0.32, *p* < 0.001). No significant relationship between total NS scores and education years was found (*r* = 0.13; *p* > 0.05). Furthermore, males and females did not differ in terms of total NS scores (*t* = −0.02; *p* > 0.05).

There was no significant relationship between total HA scores and age (*r* = 0.15; *p* > 0.05) or education years (*r* = −0.070; *p* > 0.05). Females scored significantly higher on HA (total and HA1, HA2, and HA3) than males (HA: *t* = 3.51; *p* < 0.005; HA1: *t* = 3.05, *p* < 0.005; HA2: *t* = 3.58, *p* < 0.005; HA3: *t* = 2.14, *p* < 0.05; HA4: *t* = 1.93, *p* > 0.05).

No significant relationship between total RD scores and age was found (*r* = 0.10; *p* > 0.05). There was a significant positive relationship between RD scores (RD3 and RD4) and education years (RD3: *r* = 0.25, *p* < 0.05; RD4: *r* = 0.27, *p* < 0.05). Females scored significantly higher on RD1 than males (*t* = 2.32; *p* < 0.05).

No significant relationship was found between *P* scores and age (*r* = 0.03; *p* > 0.05) or education years (*r* = −0.04; *p* > 0.05). Furthermore, males and females did not differ in terms of P scores (*t* = −1.17; *p* > 0.05).

### Relationships between TCI scores and cerebellar structures

#### VBM analyses

No significant association between WM volumes and NS scores was found.

Significant positive associations between GM volumes in left lobule VIII and NS scores (total and NS2 and NS3) were found. Furthermore, significant positive relationship between GM volumes in right lobules VIIb and VIII and left Crus 2 and NS3 scores was found (Table 1; Figure 1; Supplementary Materials, Figure 1).

**Table 1:**
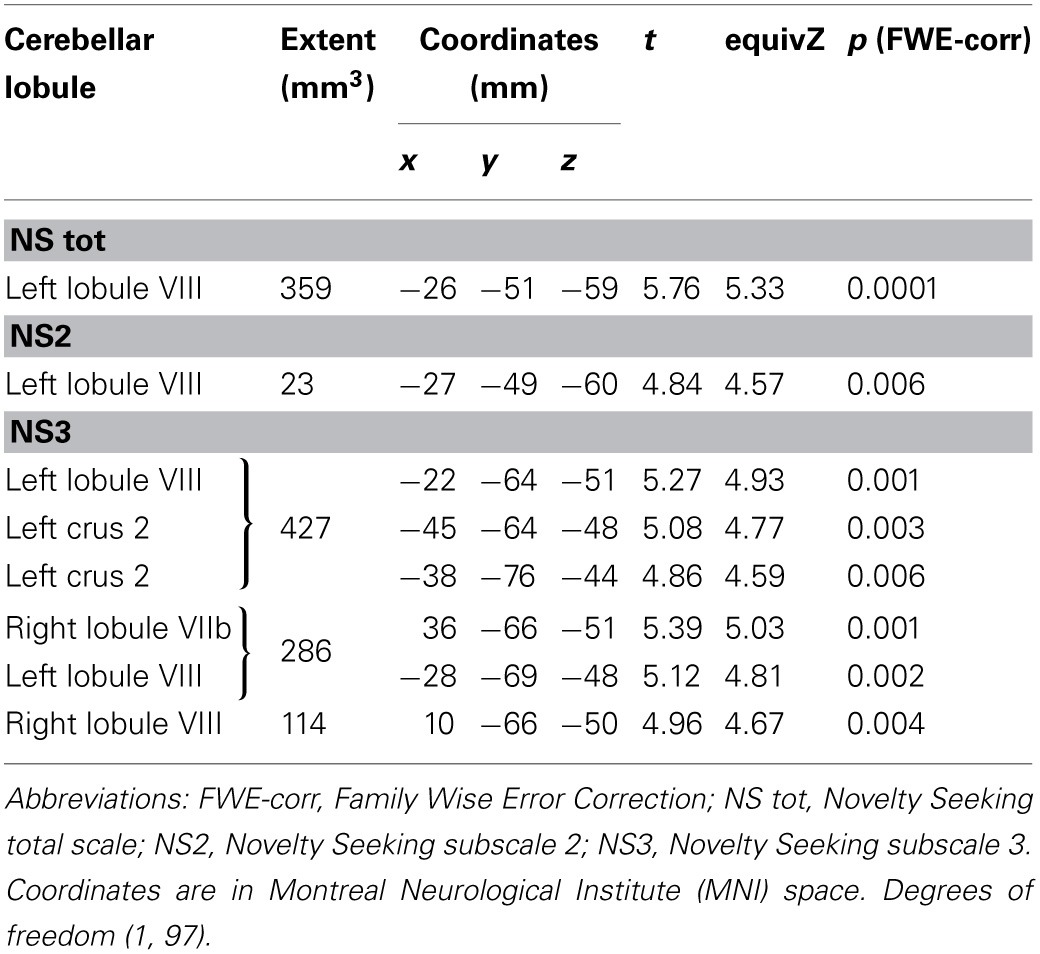
**Positive association between cerebellar gray matter volume and Novelty Seeking**.

Abbreviations: FWE-corr, Family Wise Error Correction; NS tot, Novelty Seeking total scale; NS2, Novelty Seeking subscale 2; NS3, Novelty Seeking subscale 3.

Coordinates are in Montreal Neurological Institute (MNI) space. Degrees of freedom (1, 97).

No significant associations between GM or WM volumes and HA, RD, and *P* scores were found. However, given that in our previous dataset a negative association between GM and WM volumes and HA scores was found (Laricchiuta et al., 2012), for mere confirmatory purposes, we lowered the statistical threshold at *p* < 0.0001 (uncorrected for FWE multiple comparisons). An inverse relationship between HA scores and GM and WM volumes was found in two small clusters (12 and 9 voxels) in the right lobule VIII and in the left lobule IV, respectively.

#### DTI analyses

MD values were negatively associated with NS scores (total and NS1, NS3, NS4). Namely, MD values in right lobule VI were negatively associated with NS total and NS1 scores; MD values in right lobules IV–V and Crus 1 as well as left lobules IV, V, VI, IX and Crus 2 were negatively associated with NS3 scores; MD values in right lobule VI and left lobule VIII were negatively associated with NS4 scores (Table 2; Figure 2; Supplementary Materials, Figure 2). Noteworthy, NS scores positively associated with GM volumes and negatively with GM MD emphasized that the increase of cerebellar volumes was associated with a decrease of water diffusion mobility and increase of structural density.

**Table 2:**
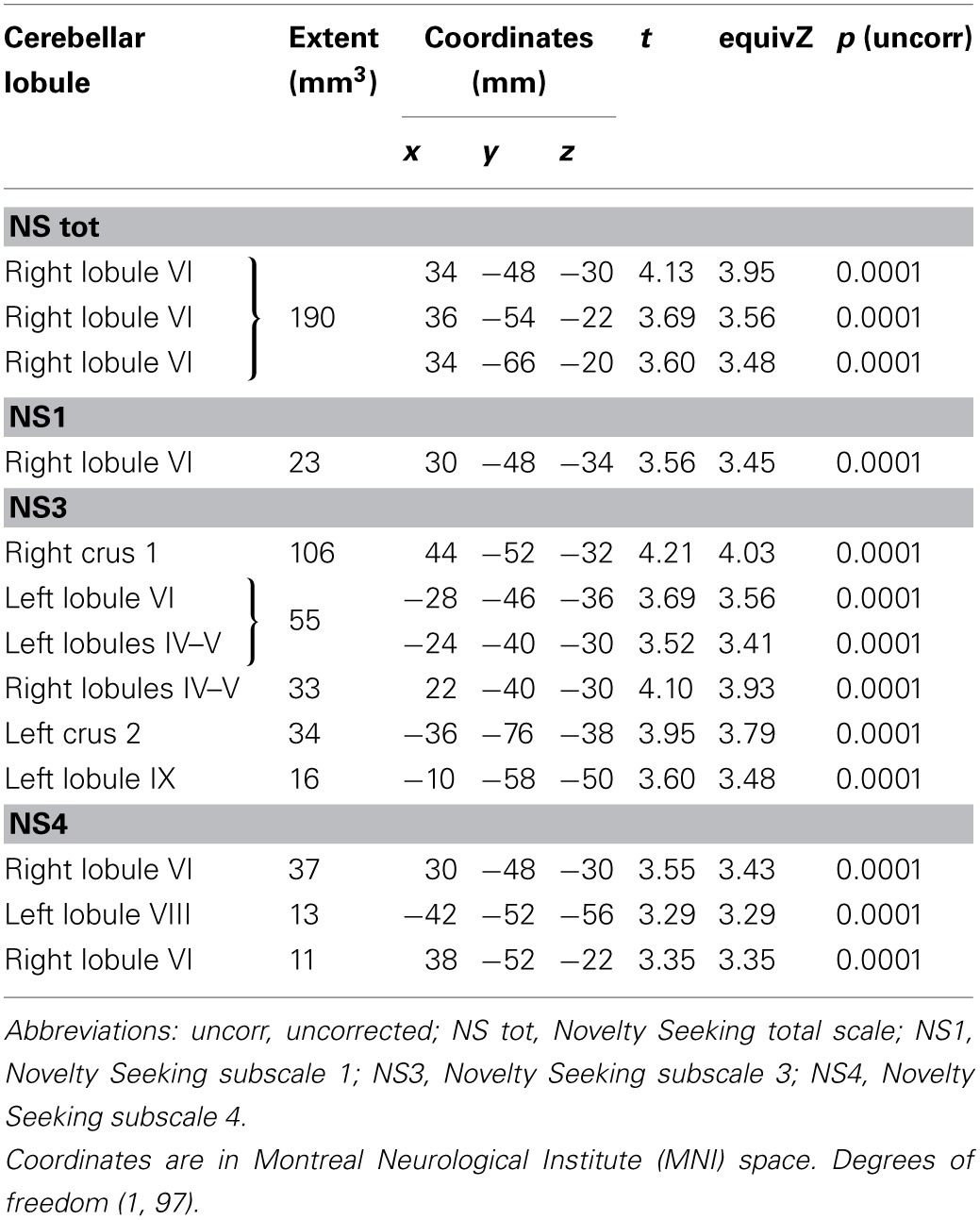
**Inverse association between cerebellar gray matter Mean Diffusivity and Novelty Seeking**.

Abbreviations: uncorr, uncorrected; NS tot, Novelty Seeking total scale; NS1, Novelty Seeking subscale 1; NS3, Novelty Seeking subscale 3; NS4, Novelty Seeking subscale 4.

Coordinates are in Montreal Neurological Institute (MNI) space. Degrees of freedom (1, 97).

FA values showed a significant positive association with NS (total and NS1, NS2, NS3) scores. Namely, lobules IV, V, and lobule VI were positively associated with NS total, NS1 and NS2 scores; right lobules IV, V, and VI as well as left lobules IV, V, and IX were positively associated with NS3 scores (Table 3; Figure 3; Supplementary Materials, Figure 3).

**Table 3:**
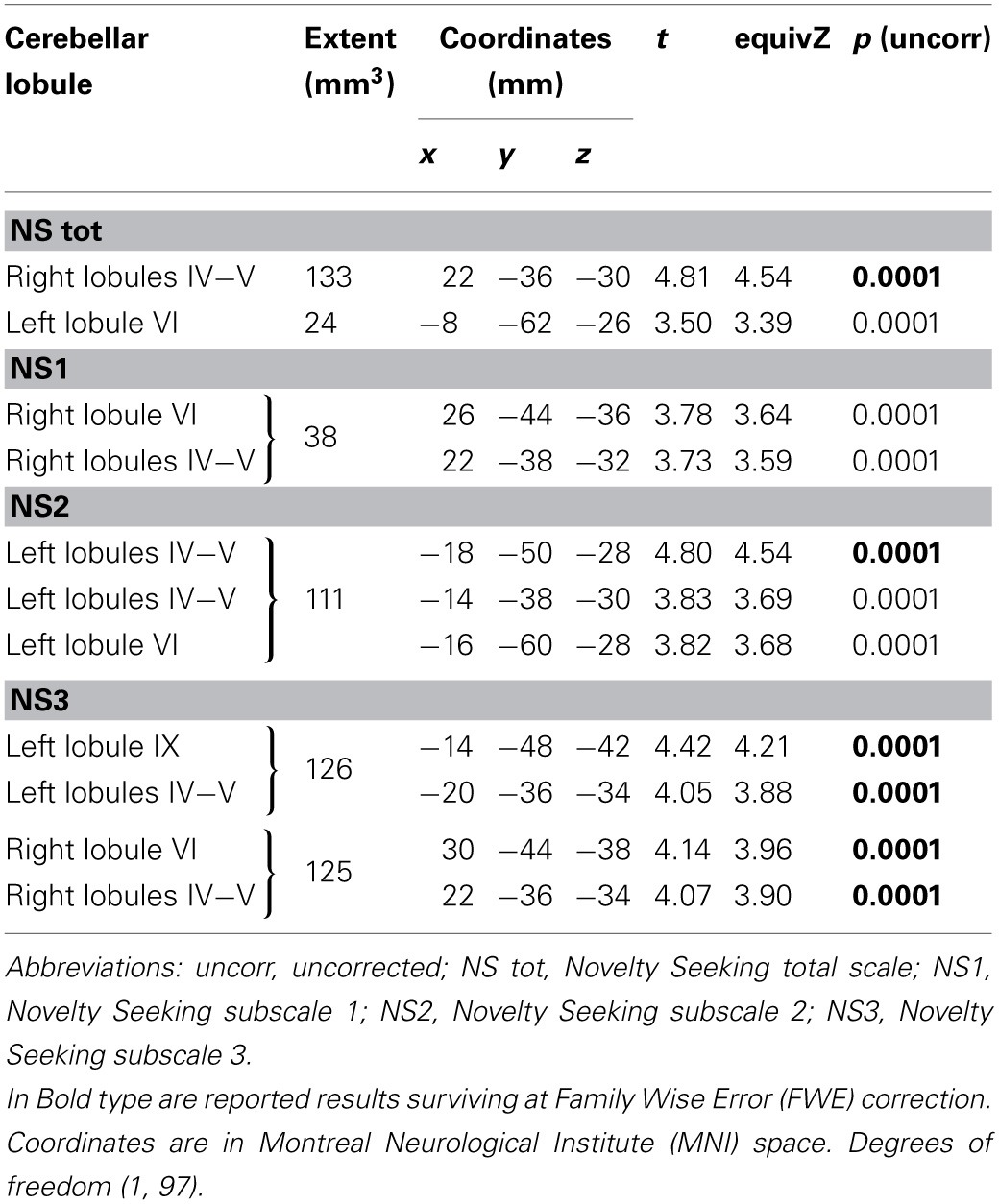
**Positive association between cerebellar white matter Fractional Anisotropy and Novelty Seeking**.

Abbreviations: uncorr, uncorrected; NS tot, Novelty Seeking total scale; NS1, Novelty Seeking subscale 1; NS2, Novelty Seeking subscale 2; NS3, Novelty Seeking subscale 3.

In Bold type are reported results surviving at Family Wise Error (FWE) correction.

Coordinates are in Montreal Neurological Institute (MNI) space. Degrees of freedom (1, 97).

No significant associations between DTI indices and HA, RD, and *P* scores (total and sub-scales) were found.

## Discussion

In the present research significant associations between the NS trait and the macro- and micro-structural indices of specific cerebellar GM and WM areas were found. Conversely, no significant associations with HA, RD, and *P* traits were evidenced. In our preceding study we found that NS was positively and HA negatively associated with WM and GM cerebellar volumes (Laricchiuta et al., 2012). Essentially, regardless the methodology used (ROI-based vs. voxel-based), in both researches we evidenced a positive relation between cerebellar structures and NS scores. Conversely, while in the previous report the negative relation between HA scores and cerebellar volumes was highly significant, in the present study such an association was found only at uncorrected statistical thresholds. Although in both studies the samples were considerably large and representative of differently aged healthy adults, the sample reduction (100 vs. 125 subjects) and variation (25 subjects participated only to the present research) as well as the different statistical analyses (FWE vs. Bonferroni's correction) required by the two neuroimaging approaches might have determined the different results. However, it is remarkable that the findings of the two studies differed only in the statistical power and not in their direction. In fact, both researches evidenced increased cerebellar volumes related to NS, and decreased cerebellar volumes related to HA.

As previously emphasized, the VBM and DTI analyses performed in the present research allowed localizing the associations between cerebellar areas and temperamental traits. We found positive associations between volumes of the vermian lobules VIIb, VIII and Crus 2 and NS scores. Notably, the limbic posterior vermis (lobules VI, VII, VIII and Crus 1 and 2) is particularly involved in the regulation of emotion and affect and in higher-level cognitive processes (Stoodley and Shmahmann, 2010), due its well-known projections to the area 46 in dorsolateral prefrontal cortex (Kelly and Strick, 2003).

The relationship between NS scores and cerebellar structures was found not only at macro- but also at micro-structural level, as indicated by MD and FA data. NS scores were associated positively with FA in lobules IV, V, VI, and IX, and negatively with MD in lobules IV, V, VI, VIII, IX, and Crus 1 and 2. In healthy subjects, a triad including increased volumes, decreased MD and increased FA indicates high integrity and efficiency, and advanced organization of the structure. Thus, the increased cerebellar GM volumes, the increased WM FA and the decreased GM MD associated with high NS scores emphasize that the macro- and micro-structural features of posterior vermis may support the behaviors of novelty-seeking.

Remarkably, the widest association between cerebellar micro-structural data and personality measures regarded levels of extravagance (NS3 sub-scale), highlighting the cerebellar involvement in the behaviors of subjects who tend to be excessive with their money, energy and feelings, to intensely feel the excitement of any new reward whereby novelty itself has reward value (Cloninger et al., 1993).

How do these findings set within the conceptual framework of the cerebellar functioning in cognitive and emotional control?

It was asserted that the cerebellum is the site where new and familiar stimuli are compared to detect discordances (Naatanen and Michie, 1979; Restuccia et al., 2007). In accordance with the cerebellar error/novelty-detection function, Ito (2008) proposed that internal models (either forward or inverse) are formed in the cerebellum to adapt motor and cognitive activities to contextual information. The internal model hypothesis for the control of novelty-related mental activities considers that the mismatch between the mental-model generated solution and the new incoming information would activate the brain novelty system. Such a system consists of the hippocampal CA1 area and midbrain dopaminergic neurons in the ventral tegmental area (VTA) (Lisman and Grace, 2005). The activation of this circuit begins when the CA1 area detects new information. The resulting novelty signal is sent via subcortical pathways passing through the ventral striatum to the VTA neurons which fire in response to novelty (Bunzeck and Düzel, 2006). The novelty system in turn would activate the attentional system during the explicit phase of novelty-related thought (Schmajuk et al., 1996). Usually, no correct solution to a new problem is derived during the explicit phase. Thus, an implicit novelty-related mental activity will be conducted by tuning the internal models encoded in the cerebellar circuitries. In triggering the new mental activity, the cerebellum could alarm the prefrontal cortex about the absence of internal models matching the novel information, maintain the newly generated internal models and incorporate them into routine schemes of thought. To successfully manage novelty, the co-activation of cerebellum and neocortical/sub-cortical areas appears then needed. The timing, prediction, and learning properties of the cerebellum, once integrated within the circuits formed with the neocortex, basal ganglia, and limbic system, could lead to the control of complex novelty-related functions (D'Angelo and Casali, 2012). Although this framework is largely speculative and without any mechanistic insight, it may be valid for interpreting the present data on the cerebellar involvement in NS trait.

Besides detection and search for novelty, a further crucial behavior of NS is the explorativity that is the proclivity to search for unfamiliar situations, making the unknown known (Minassian et al., 2011). In the present research high scores in exploratory excitability (NS1) were related with high FA and low MD in lobules IV, V, and VI. Cerebellar areas are highly involved in explorative functions that—by requiring close integration between environmental (sensory) information and searching (motor) acts—mimic the sensory-motor role classically attributed to cerebellar networks. The proneness to explorativity is mainly linked to cerebellar bidirectional interconnections with cortical and sub-cortical circuitries (Middleton and Strick, 2001; Torriero et al., 2007; Bostan et al., 2010; Cutuli et al., 2011; Foti et al., 2011; Rochefort et al., 2011). A large body of animal and human findings predicts that cerebellum, frontal cortex or both of them may be involved in actively exploring new environments. In the presence of cerebellar lesions, deficits in representing a new environment, due to inappropriate explorative pattern (Petrosini et al., 1996; Mandolesi et al., 2003) or impairment in mental folding and manipulation of tridimensional stimuli (Molinari et al., 2004) are described. Mice with mutations causing vermian hypoplasia, in particular of lobules VI–VII, exhibit reduced tendency to explore novel objects or environments and increased tendency to move about perseveratively (Caston et al., 1998; Fransen et al., 1998). Furthermore, cerebellar involvement in motivational drive to explore new environments as well as in motivational disorders associated with neuro-developmental pathologies has been repeatedly reported. Specifically, the reduced time spent in active exploration by autistic children has been linked to their hypoplasia of vermian lobules VI–VII (Pierce and Courchesne, 2001). The impairment in exploring the environment displayed by subjects affected by Williams Syndrome has been also related to their cerebellar structural and neuro-chemical alterations (Rae et al., 1998; Foti et al., 2011; Menghini et al., 2011). Enhanced exploration frequently results in coming across new events and likewise the discovery of a new stimulus reinforces further exploration and detection of novelty. Thus, explorativity and novelty-seeking appear facets of closely related behaviors. Although not explicitly tested in the current study, a possible causal link between cerebellar structures and novelty-related behaviors appears to be suggested by these animal and human data.

Even if NS contributes to adaptive functioning, an excessive seeking for novelty may indicate the disruption of internal homeostasis by which the organism seeks to regulate its internal environment. Extreme levels of NS are characteristic of many neuropsychiatric conditions, as Attention Deficit Hyperactive Disorder (ADHD), bipolar mania, substance use disorders or schizophrenia (Bardo et al., 1996; Ende et al., 2005; Fresán et al., 2007; Loftus et al., 2008). The fronto-cerebellar connections may represent one of the biological substrates for such pathological conditions (Sim et al., 2007; Durston et al., 2011). It has been recently elucidated how the connections between cerebellum and frontal cortex could sustain the impulsivity of ADHD subjects (Merwood et al., 2012). In cocaine abusers, an enhancement of BOLD signal during tasks involving cocaine-related cues and high concentrations of norepinephrine in posterior vermis were found (Anderson et al., 2006). Furthermore, subjects affected by anti-social substance dependence had GM volumes in bilateral cerebellum and left dorso-lateral prefrontal cortex significantly smaller than controls (Dalwani et al., 2011). Although it is generally assumed that frontal lobes would underlie several features of personality, the present findings suggest a re-thinking of the way the cerebellum interacts with cerebral cortex and the kind of operations it handles.

The ability to detect and seek for novelty has been argued to be a fundamental characteristic of the mammalian nervous system (Sokolov, 1963) and the response to novel stimuli is a luxury risky but worth affording (the “lure of the unknown”; Knutson and Cooper, 2006). Perceiving sameness and emitting automatic responses enhances efficiency and reliability, but at the same time promotes premature closure, perseveration and response rigidity. As Mesulam (1998) stated, “CNS has compensated for these limitations by developing specialized neural circuits for the rapid detection of unfamiliar events.” In this sense, by loosening the rigid stimulus-response linkages, novelty-seeking behaviors represent an antithesis to the pursuit of sameness and endow the organism with rapid change and adaptation of responses. It is intriguing then that the cerebellum, and in particular the posterior vermis, is involved in the fast and flexible actions to seek novelty. Indeed, the cerebellum appears the ideal structure for supporting novelty-related behaviors. The cerebellar activity signals when sensory input differs from memory-driven expectations (that is, a sensory prediction error), guides exploratory drive in novel environments, allows a flexible switching among multiple tasks or alternatives, and makes functions faster and more adaptive (Koziol et al., 2010).

## Author contributions

Laura Petrosini, Carlo Caltagirone, Paolo Girardi, and Gianfranco Spalletta designed research; Chiara Chiapponi, Fabrizio Piras, and Eleonora Picerni performed research; Eleonora Picerni, Fabrizio Piras, Daniela Laricchiuta, Debora Cutuli, Chiara Chiapponi, andSabrina Fagioli analyzed data; all authors discussed data; Laura Petrosini, Eleonora Picerni, Daniela Laricchiuta, and Gianfranco Spalletta wrote the paper.

### Conflict of interest statement

The authors declare that the research was conducted in the absence of any commercial or financial relationships that could be construed as a potential conflict of interest.
