# Supplementary material for: New evidence for the cerebellar involvement in personality traits
Source: Front Behav Neurosci. 2013 Oct 2;7:133. doi: 10.3389/fnbeh.2013.00133 (PMC3788336; doi:10.3389/fnbeh.2013.00133)
Supplement: Supplementary file 1 [file Presentation1.PPTX]

## Slide 1
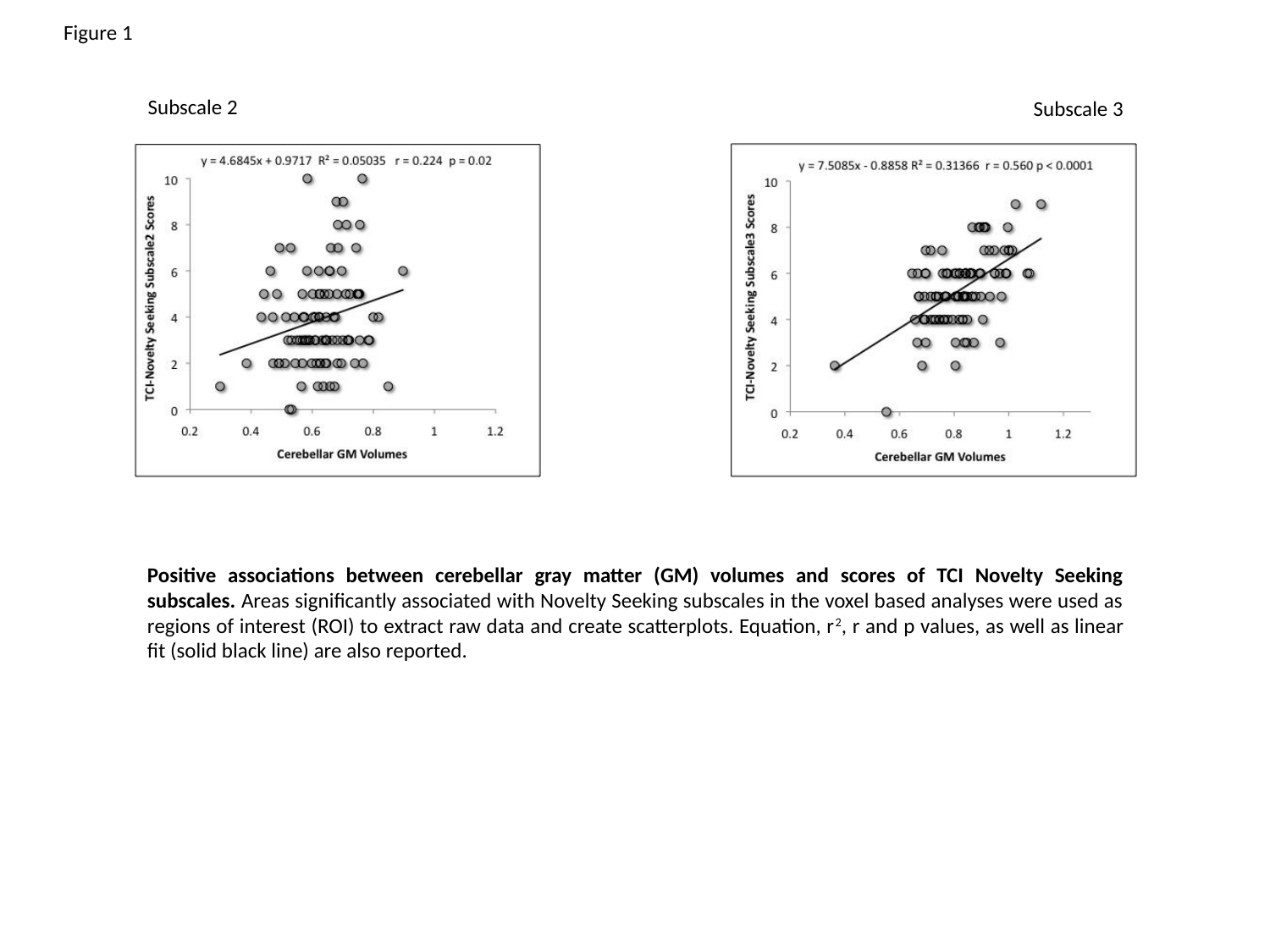

Figure 1
Subscale 2
Subscale 3
Positive associations between cerebellar gray matter (GM) volumes and scores of TCI Novelty Seeking subscales. Areas significantly associated with Novelty Seeking subscales in the voxel based analyses were used as regions of interest (ROI) to extract raw data and create scatterplots. Equation, r2, r and p values, as well as linear fit (solid black line) are also reported.

## Slide 2
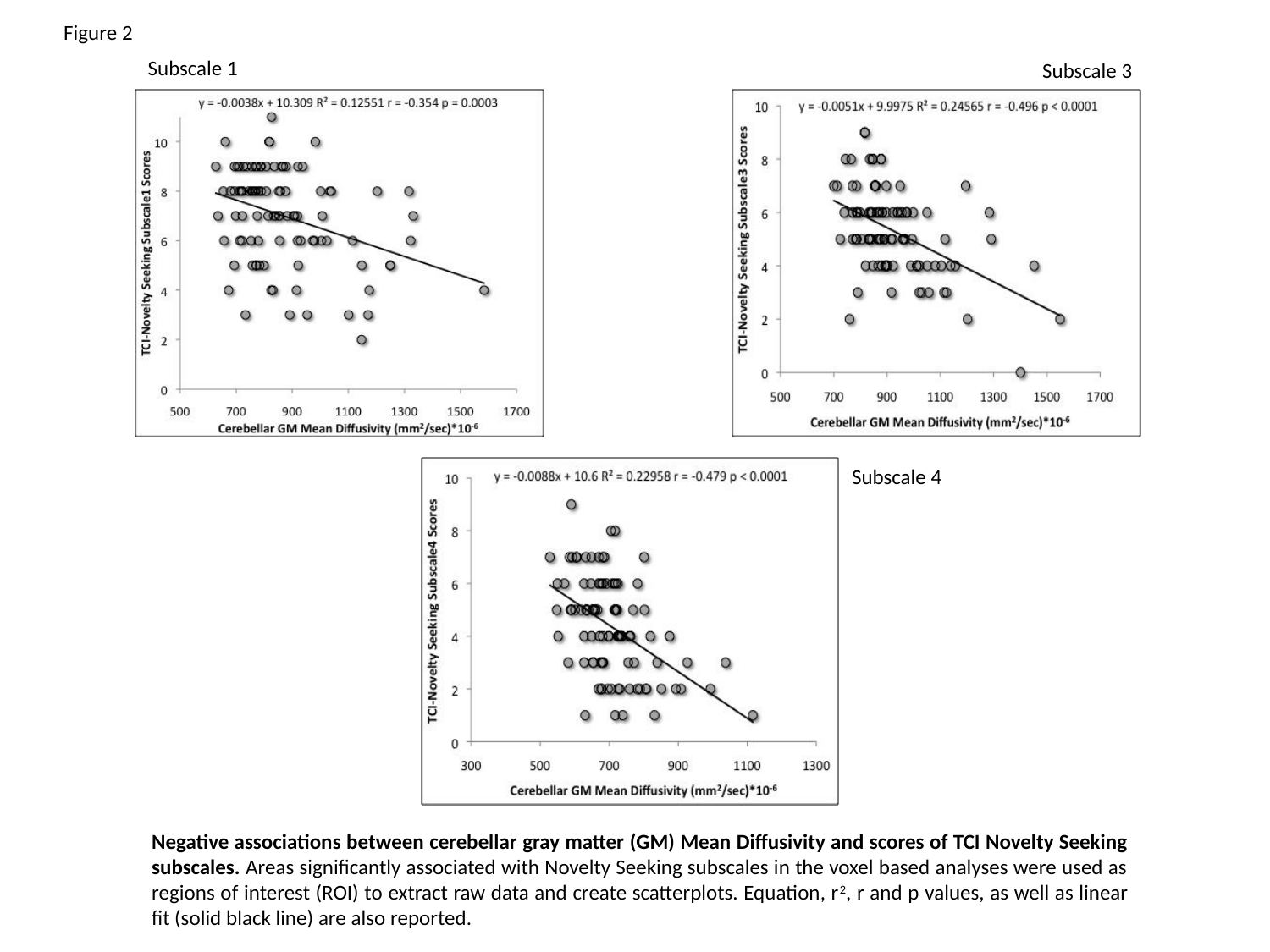

Figure 2
Subscale 1
Subscale 3
Subscale 4
Negative associations between cerebellar gray matter (GM) Mean Diffusivity and scores of TCI Novelty Seeking subscales. Areas significantly associated with Novelty Seeking subscales in the voxel based analyses were used as regions of interest (ROI) to extract raw data and create scatterplots. Equation, r2, r and p values, as well as linear fit (solid black line) are also reported.

## Slide 3
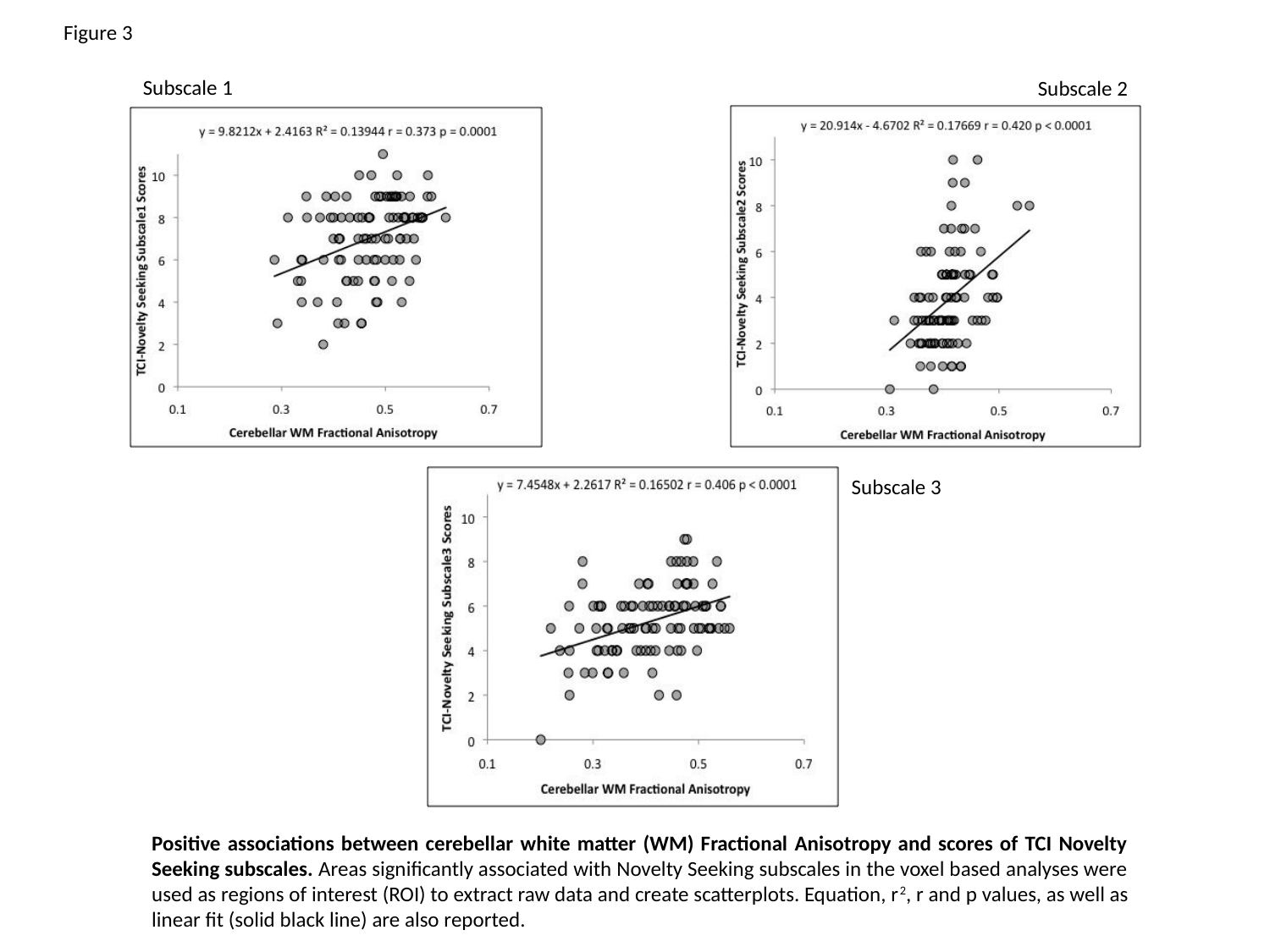

Figure 3
Subscale 1
Subscale 2
Subscale 3
Positive associations between cerebellar white matter (WM) Fractional Anisotropy and scores of TCI Novelty Seeking subscales. Areas significantly associated with Novelty Seeking subscales in the voxel based analyses were used as regions of interest (ROI) to extract raw data and create scatterplots. Equation, r2, r and p values, as well as linear fit (solid black line) are also reported.
